# WHO’S ON YOUR TEAM? DEMENTIA FAMILY NETWORKS AND CAREGIVER WELL-BEING

**DOI:** 10.1093/geroni/igad104.3697

**Published:** 2023-12-21

**Authors:** Amanda Leggett, Srabani Haldar, Sophia Tsuker, HwaJung Choi

**Affiliations:** Wayne State University, Detroit, Michigan, United States; Wayne State University, Detroit, Michigan, United States; Wayne State University, Detroit, Michigan, United States; University of Michigan, Ann Arbor, Michigan, United States

## Abstract

While caregivers are typically enmeshed in broad networks of family and friends assisting with care, this network is largely neglected, in favor of examining a “primary” caregiver. This study examines types of family/unpaid friend networks for persons living with dementia and how one’s network type relates to caregiver well-being. Data are drawn from the nationally representative 2017 National Health and Aging Trends Study and associated National Study of Caregiving. The sample includes 336 dementia care networks (Network size mean=2.9). We first identified network types using Latent Class Analysis and then examined the extent to which network type is associated with outcomes of caregiver well-being (emotional difficulty, overload) and support (social/care-related support from family and friends) using ANOVA and linear regressions adjusting for caregiver demographics. The three network types identified were: “Specialists”- small networks with little task sharing between members (29.8% of networks), “Small but mighty”- small networks with high task sharing (23.0% of networks), and “Sharers”- large networks with diverse membership and members who share and who specialize in task assistance (47.2%). “Small but mighty” network membership was associated with significantly greater emotional difficulty of caregiving (F=4.0, p<.05) and marginally greater overload (F=2.8, p=.06) than “Specialist” and “Sharer” membership. However, in regressions, only social support significantly differed, with “Sharers” (B=-0.1, p<.01) and “Small but mighty” (B=-.2, p<.05) networks receiving significantly more support than “Specialists”. Networks with more task sharing may require more social support, yet smaller network size, while carrying complex caregiving responsibilities, may take a greater emotional toll.

